# Risk of food insecurity in undocumented migrant households in Birmingham, UK

**DOI:** 10.1093/pubmed/fdab408

**Published:** 2022-01-18

**Authors:** Andrew Jolly, Janice L Thompson

**Affiliations:** School of Health Professions, University of Plymouth, Drake Circus, Plymouth, PL4 8AA, UK; School of Sport, Exercise and Rehabilitation Sciences, University of Birmingham, Edgbaston, Birmingham, B15 2TT, UK

**Keywords:** food security, migration, poverty

## Abstract

**Background:**

This study aimed to understand the extent of household food insecurity amongst undocumented migrant families in Birmingham,UK.

**Methods:**

Cross-sectional survey of households (*n* = 74) with dependent children using the USDA 18-item household food security (HFS) module. All households had an irregular immigration status and were accessing an immigration advice drop-in service (*n* = 98 adults; *n* = 138 children) in Birmingham.

**Results:**

About 95.9% of households were food insecure, and 94.6% of children lived in households with low or very low food security. Food insecurity varied within households. Around 91.8% of adults were food insecure, compared to 75.6% of children. Spearman’s rank-order correlation indicated a statistically significant positive correlation between household food insecurity level and number of children (rho = 0.253, *P* = 0.031). A Kruskal–Wallis H Test indicated no statistically significant difference (*P* = 0.730) in HFS score between households supported by asylum support, children’s social services or paid employment in the informal economy and those that had no regular income.

**Conclusions:**

Prevalence of HFS was higher in this sample of undocumented migrant households with dependent children in Birmingham, UK, than in the wider population, and larger households were more food insecure. Households without a regular income were no more likely to be food insecure than households with financial support.

## Introduction

Over the past decade there has been renewed interest in food insecurity in the UK, with a small but growing academic literature on food insecurity and the rise of emergency food aid in both the UK^1–3^ and across Europe^4^ since 2010. Food insecurity is associated with poorer access to healthcare in children,^5^ increased risk of respiratory illnesses in children^6^^,^^7^ and increased risk of depression and stress in adults.^8^ With the onset of the coronavirus disease of 2019 (COVID-19) pandemic, concerns have been raised about the expected increase in food insecurity in both the global north and global south,^9^^,^^10^ with evidence that demand for food banks was beginning to exceed supply in the UK.^11^

Although there is evidence that food aid is underutilized by religious and ethnic minorities in the UK^12^ and that lone parents and large families are at greater risk of food insecurity,^13^ there has been less attention to the prevalence of food insecurity according to immigration status. Undocumented migrants in the UK do not have the right to take up paid employment, and are subject to the no recourse to public funds (NRPF) rule, which prevents access to most mainstream social security benefits, public housing and homelessness assistance. As such, undocumented migrants in the UK, and their families, face a particular vulnerability to poverty and destitution as a result of their immigration status.^14^^,^^15^ However, there has been little research into the extent of food insecurity amongst undocumented migrants in theUK.

This paper presents the results of a study conducted as part of PhD research, which aimed to identify the level of household food security (HFS) in a cohort of undocumented migrant families accessing immigration advice services in Birmingham, UK. Birmingham is a city of just over 1 million people in the West Midlands region, and is the largest UK city outside London. About 22% of Birmingham’s populations were born outside the UK, compared to 14% in the UK as a whole.^16^ The city also has high levels of deprivation. It is the most deprived local authority area in the West Midlands region, and the seventh most deprived local authority in the UK.^17^

## Methods

The USDA 18-item module was used to assess HFS in a cross-sectional survey administered at three weekly immigration advice drop-in services in Birmingham, UK. Attendees at drop-in sessions between October and December 2016 were screened for eligibility and invited to take part in the study. To be eligible, participants had to be over 18 years old, in households containing dependent children, and with an irregular immigration status either currently or at some point over the past 12 months (*n* = 81). Irregular migration status included anyone who fell into one of three categories over the previous year: illegal entrants to the UK; refused asylum seekers and people who had overstayed the length of their visa. Out of the households who were eligible at screening, 91% agreed to take part (*n* = 74 households, *n* = 98 adults and *n* = 138 children).

Results were coded into the four categories of high, marginal, low and very low food security. Participants were asked about the number of children in their household, and results were cross tabulated to identify the number and percentage of households and the number and percentage of children in each of the four levels of food security. Spearman’s rank-order correlation was used to test for correlation between household food insecurity level and size of household.

Participants were also asked for their main source of household income, and answers were coded into the following seven categories:

- Asylum support
- Children’s services
- Paid employment
- No regular support
- Charitable support
- Family
- Undisclosed

A Kruskal–Wallis H Test was used to test for statistically significant differences in HFS level between the seven household income categories.

### Ethics

Ethical approval was received from the University of Birmingham Research Ethics Committee (ERN_15-1390) and informed consent was obtained from all participants. Because of the vulnerable nature of the group and the likelihood that participants would be food insecure, research incentives were provided in the form of supermarket vouchers, and bus passes were provided to cover travel costs on the day. Anonymity was ensured by assigning a unique code to each participant, which was kept in a separate codebook to prevent identification.

## Results

**Fig. 1:**
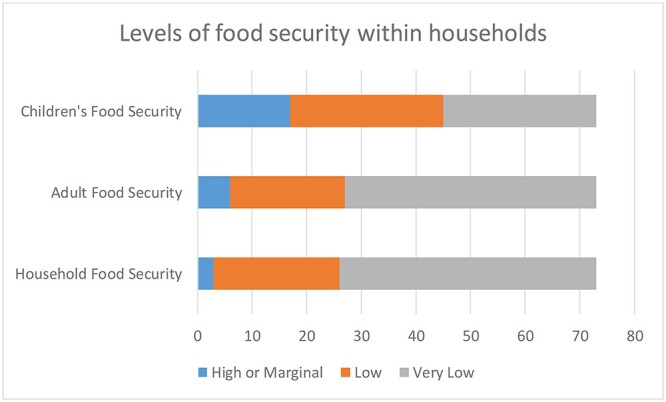
Differing levels of food security between children and adults within households accessing an immigration advice drop-in service in Birmingham, UK.

Overall, 95.9% of households were food insecure, and 94.6% of children lived in households that were food insecure at a household level, with 63.5% living in households that had very low food security, defined by the USDA as a situation where: at times during the year, the food intake of household members was reduced and their normal eating patterns were disrupted because the household lacked money and other resources for food.^18^ Just over 4% lived in families that were food secure (See Table 1). Within households, there were generational differences in the level of food insecurity, with adults being more likely to be food insecure than children within the same household. About 91.8% of adults were food insecure, compared to 75.6% of children (see Fig. 1).

**Table 1:** HFS levels of 74 households accessing an immigration advice drop-in service in Birmingham,UK

Level of household food security	Households		Children in households	
	Number in full sample (*n*)	% in full sample	Number in full sample	% in full sample
High	3	4.05	2	1.45
Marginal	1	1.35	2	1.45
Low	23	31.08	36	26.08
Very low	47	63.51	98	71.01

Spearman’s rank-order correlation indicated a statistically significant positive correlation between household food insecurity level and number of children in the household (rho = 0.253, *P* = 0.031).

Sixty-three of the 74 households disclosed their main source of income (see Table 2). A Kruskal–Wallis H Test indicated no statistically significant difference (*P* = 0.730) in HFS score between households supported by asylum support, social services and paid employment in the informal economy, or those that reported no regular income. Those receiving support from friends and family had a lower mean HFS raw score (lower food insecurity) than those receiving either form of government support, but this difference was not statistically significant.

**Table 2:** Mean rank scores and Kruskal–Wallis H test results for HFS level of households accessing an immigration advice drop-in service in Birmingham, UK across various sources of income

	Income source	*N*	Mean rank^*^
HFS level	No regular support	3	43.50
	Charitable support	1	43.50
	Paid employment	9	33.50
	Asylum support	11	32.59
	Children’s services	37	30.41
	Family	2	28.50
	Total	63	
**Kruskal–Wallis H**			2.807
**df**			5
**Asymp. Sig.**			0.730

^*^A higher mean rank score indicates higher levels of food insecurity.

## Discussion

### Main finding of this study

The prevalence of food insecurity in households that participated in the study was sixteen times higher than the rate of food insecurity in households in England, Wales and Northern Ireland. According to Wave 4 of the Food Standards Agency ‘Food and You’ household survey, household food insecurity in 2016 was 6%.^19^ Food insecurity in the present study was also higher than for other low income households in the UK. It was more than four times higher than the 23% of food insecure households with incomes below £10 400 who were accessing food banks.^20^ Children in large households were more likely to be food insecure than those in smaller households.

Participants were subject to the NRPF rule because of their immigration status, and were therefore unable to access mainstream social security benefits to mitigate or prevent poverty. However, of those who disclosed financial information, a majority had access to some form of financial support, either through unofficial employment in the informal economy, help from charitable or religious institutions, friends and family or through some form of statutory support (see Table 2). Statutory support was most commonly in the form of asylum support from the UK government for those who had previously claimed asylum or ‘child in need’ support from local authority children’s social services under section 17 of the Children Act 1989. This took the form of subsistence cash payments to prevent destitution.

### What is already known on this topic?

Research from other national contexts also suggests that migrant populations are at increased risk of food insecurity. A study in California, USA, indicates that being born in Mexico and Central America was associated with higher food insecurity,^21^ and among Hispanic adults in the USA, food insecurity was highest among non-citizens.^22^ In contrast, research in Portugal found no significant difference between food security between immigrants and non-immigrants,^23^ and analysis of the National Health and Nutrition Examination Survey in the USA suggests that race/ethnicity is a greater risk factor for food insecurity than immigration.^24^

However, some research suggests that undocumented migrants have higher food insecurity than other migrants from the same country of origin. A meta-analysis of research into food security in Afghan refugees in Iran found that food insecurity was more prevalent for both undocumented migrants, and in families of larger size.^25^

Finally, evidence of the impact of government support schemes on food insecurity is also mixed. In the USA, prevalence of food insecurity in households that were accessing food stamps was significantly higher than non-participant households due to self-selection effects.^26^ However, analysis of the impact of the Personal Responsibility, Work and Reconciliation Act, which removed entitlement to federal food assistance for families containing non-citizens, suggests that the removal of entitlement to food aid explains higher levels of food insecurity experienced by non-citizen children.^27^

### What this study adds

Although there is extensive research from a US context into food security in immigrant populations, there has been less examination of this topic in the UK. The negative public health implications of the exclusion of migrants with precarious immigration statuses from health services have been well documented. Migrants are more likely to be refused healthcare, or to fear exclusion from healthcare,^28^ and more likely to wait until becoming more unwell before seeking healthcare.^29^ However, the high prevalence of food insecurity experienced by participants in this study indicate that other forms of welfare exclusions such as the NRPF rule may also contribute to poor health due to the additional risk factors associated with food insecurity.^6^^,^^7^^,^^9^

Previous research in New York City, USA, found that receiving public assistance protected against hunger amongst undocumented Mexican migrants,^30^ but this finding was not replicated in the present study sample. There was no statistically significant difference in HFS between those receiving support and those who were not. This suggests that levels of subsistence support for migrant populations in Birmingham may not be high enough to reduce food insecurity, reflecting previous evidence that support rates under section 17 of the Children Act are below household average income poverty levels and minimum income standards.^31^

### Limitations

Although a large proportion (91%) of eligible participants took part in the study, the total sample size is relatively small, and the results are limited to participants who were accessing immigration advice services in one particular city. Additional research is needed to ascertain if the results are replicated in other geographical contexts.

Due to the hidden nature of this population and the limited reliable data about the size and demographics of the population of undocumented migrants in Birmingham, it is difficult to construct a sampling frame, and it is not possible to know how representative advice session attendees are of the numbers of undocumented households in Birmingham. The findings for this particular subset of the undocumented migrant population may not necessarily be generalizable to undocumented migrant populations in other geographic contexts.

However, as this is, to our knowledge, the first UK study of the HFS of undocumented migrants, the findings provide useful initial indicators of the extent of food security in undocumented households, to be tested with future research. Birmingham is a large city by British standards, and has both a large migrant population and high levels of deprivation, making it a particularly suitable location for this study. In addition, the type of migrant support services, which exist in Birmingham have equivalents in other UK cities, and the context of a large migrant population and high levels of deprivation is also common to other former industrial cities in the UK. As such, the findings from this study may be generalizable to other UK cities, and the approach taken can be used to inform future research studies of food insecurity in undocumented migrant households in cities with large migrant populations and high levels of deprivation.

## Conclusions

Despite the limitations of this study, the results suggest that the prevalence of food insecurity may be higher among undocumented migrant households with dependent children than other populations in the UK, is more prevalent in larger families, and is not significantly impacted by access to public assistance.

Additional research is needed to examine if these results are replicated in larger samples, different regional contexts in the UK, and in other national contexts. However, the results have implications for the levels of both asylum support and child in need support from children’s services, as they suggest that current levels of financial support offered by the Home Office and local authority children’s services are too low to mitigate the impact of food insecurity in undocumented migrant households in Birmingham, and this may be replicated in similar contexts.

## Ethical Standards Disclosure

This study was conducted according to the guidelines laid down in the Declaration of Helsinki and all procedures involving research study participants were approved by the University of Birmingham research ethics committee (ERN_15-1390). Written informed consent was obtained from all subjects.

## Data availability statement

The data underlying this article cannot be shared publicly to ensure the privacy of individuals that participated in the study.

## Notes

**Andrew Jolly**, Lecturer in Social Work

**Janice L. Thompson**, Professor of Public Health Nutrition and Exercise
